# Postural orientation and equilibrium processes associated with increased postural sway in autism spectrum disorder (ASD)

**DOI:** 10.1186/s11689-016-9178-1

**Published:** 2016-11-25

**Authors:** Zheng Wang, Rami R. Hallac, Kaitlin C. Conroy, Stormi P. White, Alex A. Kane, Amy L. Collinsworth, John A. Sweeney, Matthew W. Mosconi

**Affiliations:** 1 Schiefelbusch Institute for Life Span Studies and Clinical Child Psychology Program, University of Kansas, 1000 Sunnyside Ave., Suite 2004, Lawrence, KS 66045 USA; 2 Kansas Center for Autism Research and Training (KCART), University of Kansas Medical School, Overland Park, KS 66213 USA; 3 Analytical Imaging and Modeling Center, Children’s Medical Center, Dallas, TX 75235 USA; 4 Center for Autism and Developmental Disabilities, University of Texas Southwestern Medical Center, Dallas, TX 75390 USA; 5 Department of Psychiatry and Behavioral Neuroscience, University of Cincinnati College of Medicine, Cincinnati, OH 45219 USA

**Keywords:** Autism spectrum disorder, Postural orientation, Postural equilibrium, Static and dynamic stances, Virtual time-to-contact, Mutual information

## Abstract

**Background:**

Increased postural sway has been repeatedly documented in children with autism spectrum disorder (ASD). Characterizing the control processes underlying this deficit, including postural orientation and equilibrium, may provide key insights into neurophysiological mechanisms associated with ASD. Postural orientation refers to children’s ability to actively align their trunk and head with respect to their base of support, while postural equilibrium is an active process whereby children coordinate ankle dorsi-/plantar-flexion and hip abduction/adduction movements to stabilize their upper body. Dynamic engagement of each of these control processes is important for maintaining postural stability, though neither postural orientation nor equilibrium has been studied in ASD.

**Methods:**

Twenty-two children with ASD and 21 age and performance IQ-matched typically developing (TD) controls completed three standing tests. During static stance, participants were instructed to stand as still as possible. During dynamic stances, participants swayed at a comfortable speed and magnitude in either anterior-posterior (AP) or mediolateral (ML) directions. The center of pressure (COP) standard deviation and trajectory length were examined to determine if children with ASD showed increased postural sway. Postural orientation was assessed using a novel virtual time-to-contact (VTC) approach that characterized spatiotemporal dimensions of children’s postural sway (i.e., body alignment) relative to their postural limitation boundary, defined as the maximum extent to which each child could sway in each direction. Postural equilibrium was quantified by evaluating the amount of shared or mutual information of COP time series measured along the AP and ML directions.

**Results:**

Consistent with prior studies, children with ASD showed increased postural sway during both static and dynamic stances relative to TD children. In regard to postural orientation processes, children with ASD demonstrated reduced spatial perception of their postural limitation boundary towards target directions and reduced time to correct this error during dynamic postural sways but not during static stance. Regarding postural equilibrium, they showed a compromised ability to decouple ankle dorsi-/plantar-flexion and hip abduction/adduction processes during dynamic stances.

**Conclusions:**

These results suggest that deficits in both postural orientation and equilibrium processes contribute to reduced postural stability in ASD. Specifically, increased postural sway in ASD appears to reflect patients’ impaired perception of their body movement relative to their own postural limitation boundary as well as a reduced ability to decouple distinct ankle and hip movements to align their body during standing. Our findings that deficits in postural orientation and equilibrium are more pronounced during dynamic compared to static stances suggests that the increased demands of everyday activities in which children must dynamically shift their COP involve more severe postural control deficits in ASD relative to static stance conditions that often are studied. Systematic assessment of dynamic postural control processes in ASD may provide important insights into new treatment targets and neurodevelopmental mechanisms.

**Electronic supplementary material:**

The online version of this article (doi:10.1186/s11689-016-9178-1) contains supplementary material, which is available to authorized users.

## Background

Sensorimotor deficits are common in children with autism spectrum disorder (ASD) [1, 2]. Gross motor activities, including walking, jumping, and running, appear to be disrupted in ASD across development [3–5]. Postural control is a fundamental gross motor skill critical for stabilizing trunk orientation and executing upper and lower limb movements [6–8]. Reduced postural stability has been repeatedly demonstrated in ASD, and it appears to interfere with the development of fine motor skills [9, 10] and patients’ ability to coordinate behavior during social interactions [2, 11]. The control mechanisms underlying increased postural sway in ASD have not been established. The present study aimed to determine the control processes contributing to increased postural sway in ASD in order to identify new targets for treatments aimed at increasing postural stability in affected children and to better understand neurodevelopmental mechanisms associated with atypical sensorimotor behaviors in ASD.

**Fig. 1:**
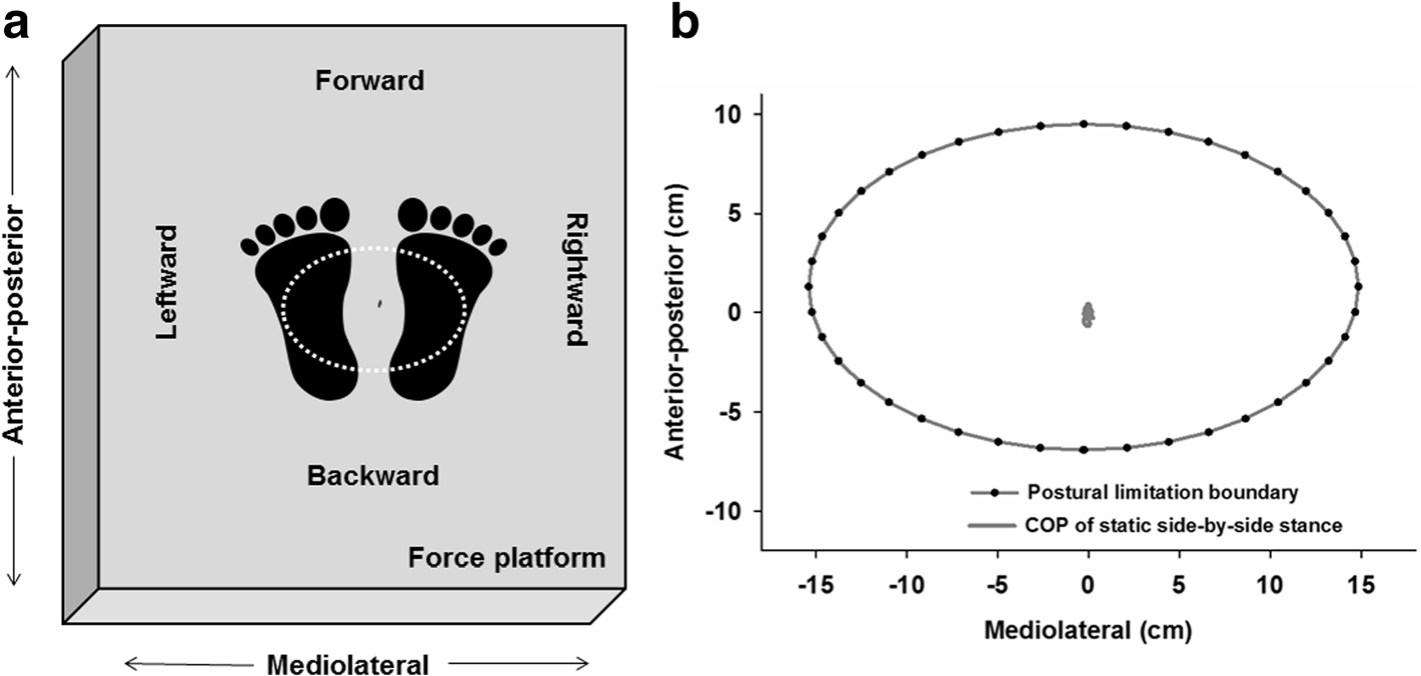
**a** Schematic representation of the spatial configuration of the force platform, a participant’s feet in the side-by-side position, the participant’s postural limitation boundary (*dashed white line*), and the COP time series (*solid gray line*) recorded during the static stance trial. All subsequent figures follow the same spatial orientation defined above. **b** Representative data from a 12-year-old TD participant showing the postural limitation boundary (*dashed black line*) and COP time series (*solid gray line*) during static stance with feet in a side-by-side position

The primary control processes used to actively support postural stability include both postural orientation and equilibrium [12–14]. Postural orientation involves active alignment of the trunk and head with respect to support surfaces, which is a process relying on multi-sensory processing, reweighting and integration. Previous studies have indicated that reductions in postural stability among children with ASD are more severe under conditions in which sensory information is occluded or degraded [4, 6, 15–18] suggesting that postural orientation processes may be disrupted in affected children. Studies of postural stability under different sensory conditions provide only indirect measurements of postural orientation processes, however, as they do not specify spatial or temporal characteristics of children’ sway relative to their support surface. A virtual time-to-contact (VTC) approach has been used repeatedly in motor control and biomechanical studies to provide more direct measurements of postural orientation [19–23]. This approach quantifies the relationship in both spatial (VTC (*ω*)_Spatial_) and temporal (VTC (*τ*)_Temporal_) domains between children’ postural sway relative to their own postural limitation boundary—i.e., the maximum extent to which children’s center of pressure (COP) can travel without their losing balance [12, 21, 23] (Fig. 1).

To maintain stability, children must instantaneously perceive spatial information regarding their postural sway relative to their own postural limitation boundary and then adjust their sway promptly to avoid moving closer to their boundary and losing balance [12, 19–23]. Multiple prior studies have shown that interpreting postural sway information is confounded if it is not measured in the context of an individual’s postural limitation boundary [21–23]. For example, patients with Parkinson’s disease show reduced COP variability in both anterior-posterior (AP) and mediolateral (ML) directions during static stance, but they also have smaller postural limitation boundaries rendering a less stable posture as their COP range of motion relative to their boundaries is significantly greater compared to healthy adults [20]. VTC measurements thus provide quantitative spatial and temporal information regarding the active orientation processes children use to align their body sway during stance.

Postural equilibrium involves the coordination of joint movements to stabilize body motion during both static and self-initiated dynamic stances. Postural sway in the AP direction (COP_AP_) is predominantly controlled by anti-gravity torques generated from the dorsi-/plantar-flexion of the ankle joints while ML sway (COP_ML_) is controlled by hip abduction/adduction movements involving sideward shifts of body weight [12, 24–26]. These two distinct joint movements show moderate dependency when healthy individuals attempt to remain still while standing indicating that a certain amount of shared information between ankle and hip joint movements is necessary for individuals to maintain balance. In patients with neurodegenerative disorders or when healthy participants close their eyes, a significant increase in shared information has been observed indicating an increased dependency of ankle and hip coordination when postural control systems are compromised [27] or under challenging conditions [26]. On the contrary, during self-initiated dynamic stances in which participants’ sway is targeted in one direction (i.e., either the AP or ML direction), a reduction in shared information is expected. For example, the ideal COP movement during intentional AP sway would be a straight line moving along the target direction without any lateral deviation indicating a minimum involvement of hip abduction/adduction or sideways body weight shifting. No known studies have examined the dependency of ankle and hip coordination in children with ASD.

The majority of postural control studies in ASD have examined static stances in which children attempt to minimize movement of their COP while standing still [4, 8, 15–18, 28]. These studies have documented increases in sway and sway variability that are associated with worse clinical symptoms [29, 30]. However, dynamic stances or self-initiated postural sways in a specific direction that more closely resemble the majority of activities performed during everyday life have received less empirical attention. During a test of gait initiation (i.e., taking a step forward), Fournier et al. [28] observed reduced COP shifts to the side in children with ASD suggesting that postural control deficits contributing to reduced postural stability in ASD may be different during more dynamic stance conditions compared to static stances. The present study examined postural orientation and equilibrium in children with ASD during static stance and dynamic postural sways in both AP and ML directions. We also assessed the extent to which static and dynamic postural deficits were associated with clinical symptoms in ASD.

Our study had three aims: (1) to quantify the extent to which children with ASD showed increased postural sway during static and dynamic stances, (2) to quantify postural orientation processes in ASD by characterizing spatial and temporal dimensions of their postural sway relative to their own postural limitation boundary, and (3) to quantify postural equilibrium processes in ASD by determining the amount of shared COP_AP_ and COP_ML_ information during quiet and dynamic standing postures. For aim 1, we hypothesized that, consistent with prior studies, children with ASD would show increased COP standard deviation and trajectory length as compared with typically developing (TD) children. We also predicted that elevations in COP variability and trajectory length in ASD would be more severe during the more challenging dynamic stances compared to static stance. For aim 2, we applied the VTC approach to test the hypotheses that children with ASD would show a compromised ability to actively acquire spatial information regarding the direction of their body sway, and reduced time to correct their movement prior to approaching their postural limitation boundary. For aim 3, we predicted that children with ASD would show increased COP_AP_ and COP_ML_ dependency as compared to TD children during static stance and that this deficit would be more severe during dynamic stances due to the increased demands on equilibrium processes. Based on prior studies showing that motor impairments may be associated with core symptoms of ASD [2, 7, 11, 29, 30], we also hypothesized that postural sway deficits in children with ASD would be related to the severity of their ASD symptoms, including clinically rated social-communication abnormalities and repetitive behaviors.

## Methods

### Participants

Twenty-two children with ASD and 21 age, height, weight, sex, and performance IQ-matched TD children completed tests of static and dynamic stances (Table 1). IQ was assessed using the Wechsler Abbreviated Scales of Intelligence [31] for all participants except for one younger participant who completed the Wechsler Preschool and Primary Scale of Intelligence—Fourth Edition [32]. Children with ASD were recruited through community advertisements and local clinical programs. The diagnosis of ASD was established with the Autism Diagnostic Inventory-Revised (ADI-R) [33], the Autism Diagnostic Observation Schedule—II (ADOS-II) [34], and expert clinical opinion based on DSM-V criteria. Potential patients were excluded if they had any known genetic condition associated with ASD. TD participants were recruited from the community and were required to have a score of 8 or lower on the Social Communication Questionnaire (SCQ) [35]. Potential TD participants were excluded for current or past psychiatric or neurological disorders; family history of ASD in first-, second-, or third-degree relatives; or a history in first-degree relatives of a developmental or learning disorder, psychosis, or obsessive compulsive disorder based on a brief screening interview.

**Table 1:** Demographic characteristics [mean (SD)] of children with ASD and typically developing (TD) children

	ASD (*n* = 22)	TD (*n* = 21)	*t*	*p*
Age (years)	12.72 (3.64)	11.67 (4.53)	0.719	0.401
Range	7–18 years	4–18 years		
Height (cm)	154.3 (24.45)	142.90 (23.09)	2.493	0.122
Weight (kg)	55.00 (27.54)	41.77 (20.88)	3.123	0.085
% male^a^	86.4%	85.7%	0.004	0.951
FSIQ^b^	98.68 (17.15)	108.05 (14.29)	3.766	0.059
Range	70–131	80–141		
PIQ	103.45 (16.74)	104.24 (12.69)	0.030	0.864
Range	72–132	80–129		
VIQ	94.50 (18.01)	109.81 (15.26)	9.006	0.005**
Range	64–129	85–129		

*FSIQ* full-scale IQ, *PIQ* performance IQ, *VIQ* verbal IQ

Statistical significance at ** *α* = 0.01

^a^Chi-square (*χ* ^2^) statistics

^b^Full-scale IQ shows marginal statistical significance

No participants were taking medications known to affect motor performance at the time of testing, including antipsychotics, stimulants, or anticonvulsants [36]. Four children with ASD were taking antidepressant medications and two were taking antihypertensive medications at the time of testing. No participant had a history of head injury, birth asphyxia, or non-febrile seizure. Participants 18 years of age or older provided written consent and minors provided assent in addition to written consent from their legal guardian. Study procedures were approved by the local Institutional Review Board.

### Apparatus and procedures

Postural testing was conducted for children with ASD either during children’s first visit after the completion of their clinical assessment or during a second visit that was no later than three weeks from their clinical evaluation. Postural testing consisted of four experimental conditions lasting 30–40 min in total. All testing was completed with participants standing with bare feet shoulder width apart on an AMTI (American Mechanical Technology, Inc., Watertown, MA) AccuGait strain gauge force platform (size 49.78 × 49.78 cm; sampling rate of 1000 Hz).

Prior to testing, each participant’s postural limitation boundary was determined based on the maximum extent to which he/she could lean in each of four directions: forward, backward, leftward, and rightward. During the postural limitation boundary trial, participants stood with their feet side-by-side shoulder width apart, kept their arms fixed at their sides, and slowly leaned as far as possible in each of the four directions without raising their feet. Participants maintained an inclined posture while leaning for 2 s. An experimenter always observed participants’ behavioral performance and COP pattern online to ensure that they had reached their maximum extent. Participants who swayed further than their reported extent while maintaining their maximum extent for 2 s were asked to repeat the trial as done in prior studies [21, 23].

**Fig. 2:**
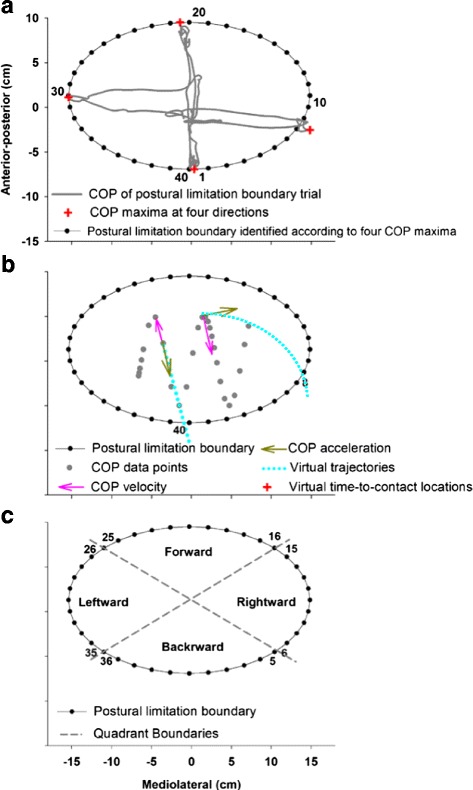
**a** The same 12-year-old TD participant’s COP time series recorded from the postural limitation boundary trial during which the participant slowly leaned forward, backward, and to each side. The maximum postural sway (*red crosses*) in each direction was used to model the postural limitation boundary (*solid black line*), which was then divided into 40 equal-sized segments (*black dots*, each with 9° expansion) identified in a counter-clockwise manner to quantify the spatial orientation of his VTC (*ω*)_Spatial_ and VTC (*τ*)_Temporal_ minima measurements. **b** Schematic representation of linear and nonlinear COP virtual trajectories (*light blue dotted lines*). The COP time series (*gray dotted line*) was amplified for demonstration purpose. The virtual trajectories were determined based on the velocity and acceleration of each COP data point (*gray dot*). The virtual trajectory has a parabolic shape if the COP data point’s initial velocity and acceleration are not co-linear (e.g., shown here intersecting with the postural limitation boundary at segment 8). The virtual trajectory is linear if the COP data point’s initial velocity and acceleration are in the same direction and either the velocity or acceleration vector is zero (e.g., shown here intersecting with the postural limitation boundary at segment 40). **c** Schematic of four quadrants defined for statistical analyses of VTC (*ω*)_Spatial_ and VTC (*τ*)_Temporal_ minima. *Numerical labels* represent the postural limitation boundary segments that were used to define quadrant in each direction. Each quadrant includes 10 postural limitation boundary segments with 90° expansions forward, backward, leftward, and rightward

Prior to the testing trial, the examiner modeled the task by leaning as far as possible in each of the four directions. Participants then were guided through the postural limitation trials by the examiner, who used the following instructions: “For the next part, you will lean your body as far as possible without raising your feet from the ground. Now, slowly lean your body forward, hold, come back to the center. Slowly lean your body backward, hold, etc.” for each of the directions. Each individual’s postural limitation boundary then was modeled as an ellipse based on the individual’s COP maximum for each direction as well as the force platform coordinates (Figs. 1 and 2a). Participants’ foot position during the postural limitation boundary trials was outlined on a tracing paper placed on top of the force platform, and children then aligned their feet with the tracings during the remaining trials so that their foot location remained constant throughout testing.

Following postural limitation boundary trials, participants completed tests of both static and dynamic stances. During the static stance test, participants were instructed to stand as still as possible on the force platform with their arms resting at their sides for three 30 s trials. During dynamic stance tests, participants completed two different self-initiated postural sways—AP and ML—each of which included three 30-s trials. For each dynamic stance condition, participants were instructed to sway continuously along the target direction at a comfortable speed and magnitude without raising their toes or heels. All trials were followed by 30 s of rest with 1 min of rest between different stance conditions. Nine postural standing trials (3 conditions × 3 trials) and one postural limitation boundary trial were examined during the test. Order of administration of the static and dynamic stance tests and the two directions of the dynamic stance test were counterbalanced across participants.

### Data processing

The initial and final 5 s of force and moment data collected from the force platform was removed from analyses in order to limit variable effects related to initiating postural stance and fatigue at the end of trials. The remaining 20 s of the force and moment time series was processed and analyzed in Matlab 2015b (MathWorks, Inc., Natick, MA). All kinetic data were down sampled to 100 Hz and low pass filtered using a fourth-order double pass Butterworth filter with a cutoff frequency of 6 Hz. The COP time series for both the postural limitation boundary trial and the standing tests were derived from force and moment data using the following formulas [37]:

1 $$ \begin{array}{c}\hfill {\mathrm{COP}}_{\mathrm{AP}}=-\frac{\left({M}_{\mathrm{ML}}-{F}_{\mathrm{AP}}\cdot {d}_z\right)}{F_z}\hfill \\ {}\hfill {\mathrm{COP}}_{\mathrm{ML}}=\frac{\left({M}_{\mathrm{AP}}-{F}_{\mathrm{ML}}\cdot {d}_z\right)}{F_z}\hfill \end{array} $$

where *F* and *M* represent ground reaction force and moment, respectively, in either the AP or ML direction; *F*_*z*_ represents the vertical ground reaction force; and *d*_*z*_ represents the offset of the force platform sensor located 7.9 mm underneath the surface of the platform.

### Data analysis

To quantify participants’ postural stability, we examined the standard deviation of each participant’s COP time series in both AP (COP_AP_) and ML (COP_ML_) directions, as well as the COP trajectory length during each trial of the static and dynamic stance tests. The COP trajectory length is estimated as the sum of the distances between consecutive points on the COP path [37]. During the dynamic stance trials, participants’ natural postural sway frequency of each trial was measured using the 20-s COP time series along the target direction (i.e., COP_AP_ was used for dynamic AP sway and COP_ML_ was used for ML sway). Due to the inherent trending of the COP data, a local maxima and minima algorithm [38] was applied to detect each sway cycle with the criteria that the range of COP movement in each direction should be >50% of the global COP range of motion of the trial. The natural sway frequency along the target direction then was calculated as follows:

2 $$ \mathrm{Freq}=\frac{\left({N}_{\max }/2\right)}{20\ \mathrm{s}} $$

where *N*_max_ stands for the number of local COP maxima detected. Due to the fact that each postural sway cycle includes two *N*_max_, natural sway frequency was calculated as (*N*_max_/2) divided by 20 s. Sway frequency was not assessed during static stance trials. All dependent variables were averaged across three trials of each condition prior to between-group comparisons.

### Virtual time-to-contact

To measure postural orientation processes, both VTC (*ω*)_Spatial_ and VTC (*τ*)_Temporal_ time series were calculated for each trial of the static and dynamic stances. The basic assumption underlying the VTC approach is that participants evaluate the spatial and temporal characteristics of their body movement relative to their postural limitation boundary, and then they use this information to adjust their COP movement away from their postural limitation boundary [12, 19–23]. VTC (*ω*)_Spatial_ quantifies the specific location at which individuals’ postural sway will collide with their postural limitation boundary under the assumption that it continues to travel along its original trajectory given its current velocity and acceleration. To determine VTC (*ω*)_Spatial_, we divided each individual’s postural limitation boundary into 40 equal-sized segments, each with 9° expansion, labeled from 1 to 40 in a counter-clockwise manner (Fig. 2a). The numeric label of the boundary segment associated with the first crossing point of the COP trajectory was assigned as VTC (*ω*)_Spatial_. Therefore, VTC (*ω*)_Spatial_ contains a time series of discrete boundary segment labels ranging from 1 to 40. The percentage distribution of VTC (*ω*)_Spatial_ at each postural limitation boundary segment relative to the total segments was examined. The distribution of VTC (*ω*)_Spatial_ increases along directions that would potentially challenge participants’ stability suggesting that children try to perceive sufficient spatial information regarding their body movement towards these “dangerous” directions and continuously use this information to evaluate their postural stability.

VTC (*τ*)_Temporal_ quantifies the time it would take for the individuals’ sway to collide with their postural limitation boundary if it continued to travel along the current trajectory at the current rate. VTC (*τ*)_Temporal_ estimates the time limit for individuals to adjust their postural sway so that their COP movement does not reach their postural limitation boundary. An increased VTC (*τ*)_Temporal_ indicates an increased amount of time for children to refine their postural movement and ensure that they do not reach their postural limitation boundary, whereas a reduced VTC (*τ*)_Temporal_ indicates that they have a reduced time to make this adjustment. The VTC (*τ*)_Temporal_ was calculated for each instantaneously measured COP data point by defining the current moment (*t*_*i*_) and estimating the virtual motion of the data point using its velocity and acceleration vectors (see ​Additional file 1 for formulas). Briefly, the resultant force and acceleration $\overrightarrow{a}\left({t}_i\right)$ were assumed to be constant while the COP data point moved along its virtual trajectory from its initial position $\overrightarrow{r}\left({t}_i\right)$ with its instantaneous initial velocity $\overrightarrow{v}\left({t}_i\right)$ until it was estimated to collide with the postural limitation boundary. The following formulas were used to calculate VTC (*τ*)_Temporal_:

3 $$ {x}_i\left(\tau \right)={r}_x\left({t}_i\right)+{v}_x\left({t}_i\right)\cdot \tau +{a}_x\left({t}_i\right)\cdot \frac{\tau^2}{2} $$

and

4 $$ {y}_i\left(\tau \right)={r}_y\left({t}_i\right)+{v}_y\left({t}_i\right)\cdot \tau +{a}_y\left({t}_i\right)\cdot \frac{\tau^2}{2} $$

where [*x*_*i*_(*τ*), *y*_*i*_(*τ*)] represents the physical contact location of VTC (*ω*)_Spatial_ at the postural limitation boundary, *r*_*x*_(*t*_*i*_) and *r*_*y*_(*t*_*i*_) are components of the instantaneous initial position vector, *v*_*x*_(*t*_*i*_) and *v*_*y*_(*t*_*i*_) are components of the instantaneous initial velocity vector, and *a*_*x*_(*t*_*i*_) and *a*_*y*_(*t*_*i*_) are components of the instantaneous initial acceleration vector in the AP and ML directions.

As Fig. 2b shows, the virtual trajectory is parabolic if the initial velocity and acceleration vectors of a COP data point are not co-linear. In this scenario, the COP data point would have multiple contacts with the postural limitation boundary at different locations. When this occurred, the minimum positive time parameter associated with the first boundary crossing (i.e., VTC (*ω*)_Spatial_) point was assigned as VTC (*τ*)_Temporal_. In contrast, the virtual trajectory is linear if a COP data point’s initial velocity and acceleration vectors have the same direction or if either of them equals zero. If both initial velocity and acceleration vectors are equal to zero, the virtual trajectory is a stationary point and VTC (*τ*)_Temporal_ is infinite because this specific COP data point will never contact the postural limitation boundary. VTC (*τ*)_Temporal_, therefore, ranges from 0 to infinity with 0 representing a COP data point that is in contact with the postural limitation boundary and infinity representing a COP data point that will never reach the postural limitation boundary given its current location, velocity, and acceleration. The minimum value of VTC (*τ*)_Temporal_ was calculated in this study as it is more informative than the maximum value [23].

### Mutual information

To examine postural equilibrium processes, the amount of mutual information shared between COP_AP_ and COP_ML_ sway was quantified. Mutual information is a measure of the dependency of two time series useful for determining the degree to which a joint distribution of *p*(COP_AP_, COP_ML_) covaries with the products of the factored marginal distributions *p*(COP_AP_) *p*(COP_ML_). Mutual information was calculated using the following formula [39]:

5 $$ MI\left({\mathrm{COP}}_{\mathrm{AP}};\ {\mathrm{COP}}_{\mathrm{ML}}\right)={\displaystyle {\int}_{{\mathrm{COP}}_{\mathrm{ML}}}}{\displaystyle {\int}_{{\mathrm{COP}}_{\mathrm{AP}}}}p\left({\mathrm{COP}}_{\mathrm{AP}},\ {\mathrm{COP}}_{\mathrm{ML}}\right){ \log}_{2\ }\left(\frac{p\left({\mathrm{COP}}_{\mathrm{AP}},\ {\mathrm{COP}}_{\mathrm{ML}}\right)}{p\left({\mathrm{COP}}_{\mathrm{AP}}\right)p\left({\mathrm{COP}}_{\mathrm{ML}}\right)}\right){d}_{{\mathrm{COP}}_{\mathrm{AP}}}{d}_{{\mathrm{COP}}_{\mathrm{ML}}} $$

where *p*(COP_AP_, COP_ML_) stands for the joint probability density function of COP_AP_ and COP_ML_, and *p*(COP_AP_) and *p*(COP_ML_) are the marginal probability density functions of COP_AP_ and COP_ML_, respectively. The unit of mutual information is bit. Higher mutual information represents more shared information across COP_AP_ and COP_ML_ time series. Given previous literature suggesting that COP_AP_ primarily is controlled by the ankle dorsi-/plantar-flexion while COP_ML_ is associated with hip abduction/adduction [24–26], our mutual information analyses allowed us to determine the extent to which distinct ankle and hip joint movements could be applied independently during standing.

### Clinical measures

The ADI-R and ADOS-II were used to examine the severity of ASD symptoms for each participant and determine the extent to which postural control deficits were associated with core symptoms. The ADI-R is a semi-structured interview conducted with parents/caregivers of children with ASD assessing early development and both past and current levels of social interaction and communication abilities as well as the presence of restricted and repetitive behaviors [33]. The ADOS-II consists of a series of structured and semi-structured tasks designed to elicit social interaction and communication behaviors that are impaired in ASD [34]. For both the ADI-R and ADOS-II, higher scores reflect more severe behavioral abnormalities in a given domain. We examined scores from the social, communication, and restricted-repetitive behavior algorithms of the ADI-R and the social-affective and restricted-repetitive behavior algorithms of the ADOS-II.

### Statistical analyses

We conducted a 3 (stance condition: static vs. AP sway vs. ML sway) × 2 (COP direction: AP vs. ML) × 2 (group: ASD vs. TD) fixed-effects repeated-measure ANOVA to examine COP standard deviation. In this model, stance condition and COP direction served as within-subject factors and group was included as a between-subject factor. A series of 3 (stance condition) × 2 (group) fixed effects repeated-measure ANOVAs were performed to assess COP trajectory length and mutual information. Stance condition was included as a within-subject factor and group was entered as a between-subject factor. Natural postural sway frequency was compared across groups during AP and ML dynamic sways only using a 2 (stance condition) × 2 (group) fixed effects repeated-measure ANOVA.

In order to quantify between-group differences on VTC (*τ*)_Temporal_ minima and VTC (*ω*)_Spatial_ relative to each individual’s postural limitation boundary, four equally spaced quadrants were defined (Fig. 2c): forward (segments 16 to 25), backward (segments 36 to 5), leftward (segments 26 to 35), and rightward (segments 6 to 15). A series of 4 (quadrants) × 2 (group) fixed-effects ANOVAs were conducted on VTC (*τ*)_Temporal_ minima and VTC (*ω*)_Spatial_ for each task condition independently. For all analyses, results were interpreted as significant if *p* < 0.05. Where Mauchly’s test indicated a violation of sphericity, the Greenhouse-Geisser correction was used to provide a conservative estimate of main and interaction effects. The normal distribution of all dependent measurements as well as homogeneity of variance between groups were examined, where no variable has shown violation of these assumptions.

Pearson correlation coefficients were used to examine the relationships between standing parameters found to be different between groups and age, IQs, and clinical ratings of ASD severity. In order to account for the large number of correlations performed for each group, a more conservative cutoff was used and results were interpreted to be significant if alpha values were less than 0.01 and correlation coefficient (*r*) was greater than 0.5.

## Results

**Fig. 3:**
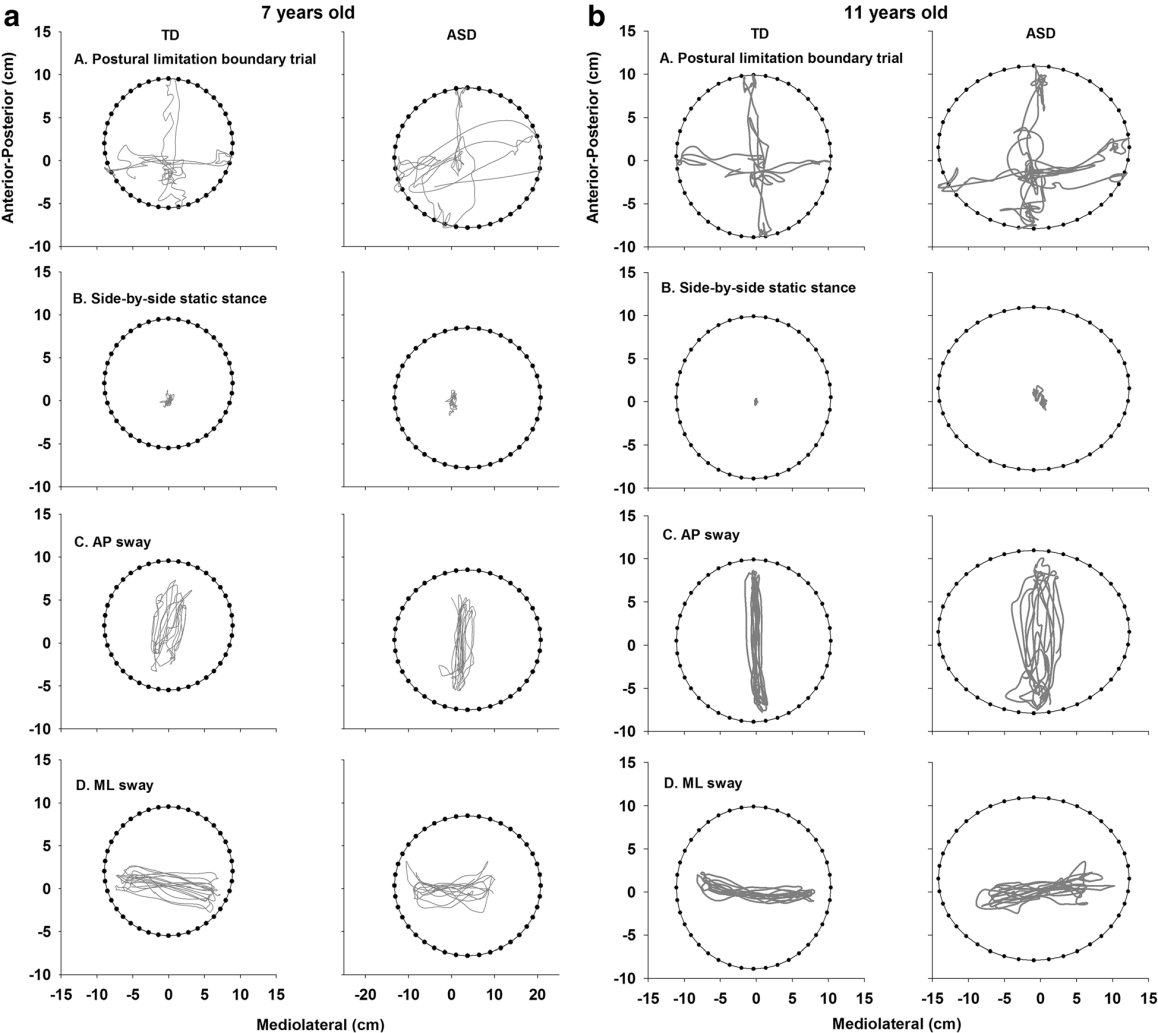
**a** Representative trials from a 7-year-old TD child (*left column*) and an age-matched ASD child (*right column*). **b** Representative trials from an 11-year-old TD (*left column*) and an age-matched ASD child (*right column*). In general, TD children show an overall COP variability reduction with age at all standing conditions while this developmental change was not observed in children with ASD. The COP time series of the ASD children shows more variability than that of their TD peers. In static stance, the children with ASD showed increased COP variability in both AP and ML directions. In both dynamic postural sway conditions, the children with ASD showed increased COP variability in the directions orthogonal to the target. For better display of the COP time series and postural limitation boundaries, scales on the *x* and *y axes* of each child’s plots were adjusted and thus are not consistent across participants. The semi-major and semi-minor axes of their postural limitation boundary were aligned with the force platform coordinate for all images

Figure 3 shows traces of the COP time series for one 7- and one 11-year-old representative control (left column) and each of their age-matched peer with ASD (right column) during the postural limitation boundary (A), static stance (B) AP sway (C) and ML sway (D) trials. Both the 7- and 11-year-old children with ASD showed increased COP variability relative to TD controls across all conditions. During static stance, increased COP variability of children with ASD was observed along both the AP and ML directions. During dynamic stances, the representative participants with ASD displayed greater COP variability than the TD children along the directions orthogonal to the targets.

**Fig. 4:**
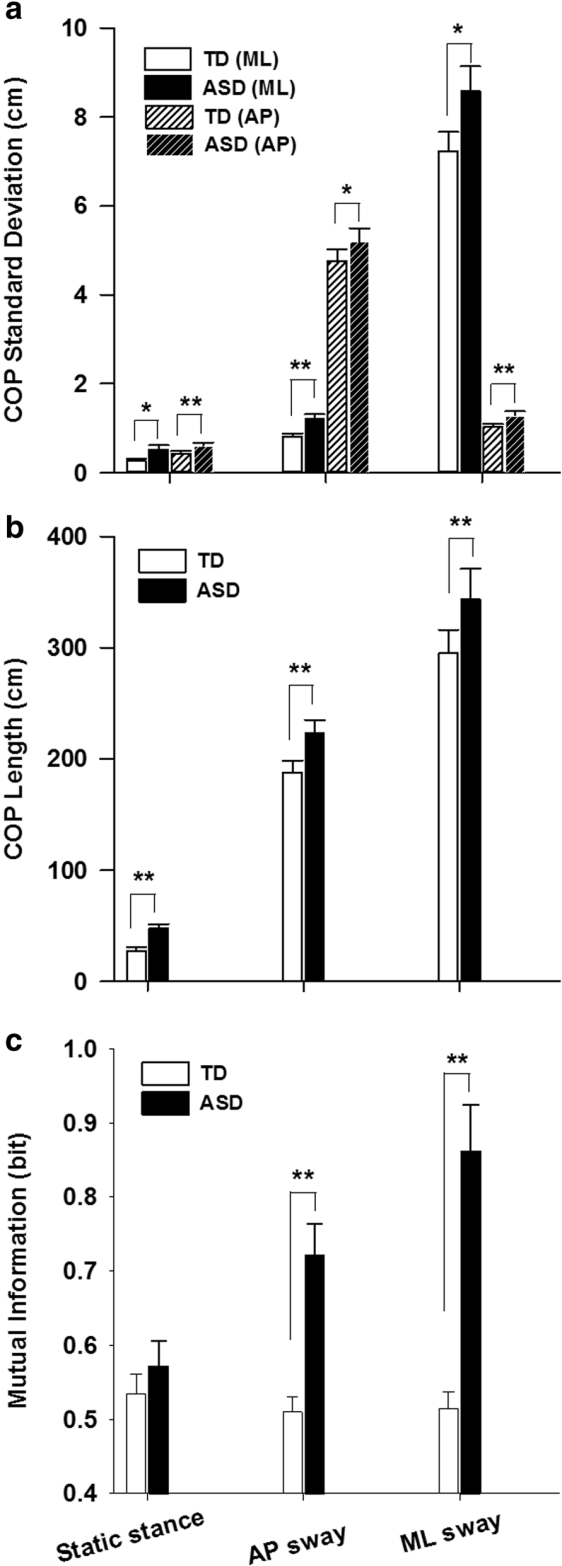
**a** COP_AP_ and COP_ML_ standard deviation. **b** COP trajectory length. **c** Mutual information shared between COP_AP_ and COP_ML_ are shown as a function of standing condition. Between-group differences are marked as *0.05 level and **0.01 level. *Error bars* represent standard error

COP standard deviation was greater for the dynamic stances compared to static stance (stance condition main effect: *F*_2, 78_ = 295.193, *p* = 0.000). COP standard deviation was greater in the AP than ML direction during static stance, whereas it was greater in the target directions during dynamic stances (condition × direction interaction effect: *F*_1.195, 46.618_ = 360.747, *p* = 0.000). Children with ASD showed increased within trial COP standard deviation compared to TD controls across all conditions and directions of postural sway (group main effect: *F*_1, 39_ = 15.347, *p* = 0.000). This difference was more severe for the dynamic stances compared to static stance (stance condition × direction × group interaction: *F*_2, 78_ = 3.198, *p* = 0.046). During static stance, children with ASD showed more severe elevations compared to TD children in COP_AP_ standard deviation relative to COP_ML_ standard deviation [ASD-TD (AP) = 0.237 cm, SE = 0.084 cm, *p* = 0.007; ASD-TD (ML) = 0.271 cm, SE = 0.112 cm, *p* = 0.020]. During dynamic stances, children with ASD showed larger differences relative to TD controls in COP standard deviation orthogonal to the target directions as opposed to along the target directions [AP sway: ASD-TD (AP) = 1.022 cm, SE = 0.495 cm, *p* = 0.046; ASD-TD (ML) = 0.464 cm, SE = 0.141 cm, *p* = 0.002; ML sway: ASD-TD (AP) = 0.303 cm, SE = 0.104 cm, *p* = 0.006; ASD-TD (ML) = 1.700 cm, SE = 0.691 cm, *p* = 0.019] (Fig. 4a).

COP trajectory length was greater for the dynamic stances compared to static stance, and for the dynamic ML sway condition compared to the AP sway condition (stance condition main effect: *F*_1.325, 51.672_ = 184.571, *p* = 0.000). Children with ASD showed greater COP trajectory length compared to TD children across all postural conditions (group main effect: *F*_1, 39_ = 8.706, *p* = 0.005). There was no significant stance condition × group interaction identified (*F*_1.325, 51.672_ = 1.272, *p* = 0.286) (Fig. 4b).

Natural postural sway frequency was similar across the two dynamic stance conditions (condition main effect: *F*_1, 40_ = 0.655, *p* = 0.423). No between-group differences were observed in terms of sway frequency (group main effect: *F*_1, 40_ = 1.482, *p* = 0.231; ASD = 0.320 Hz, SE = 0.019 Hz; TD = 0.354Hz, SE = 0.020 Hz) Additional file 2.

### Virtual time-to-contact

The postural limitation boundary area was similar for children with ASD and TD controls (*t*_41_ = 0.482, *p* = 0.632; ASD = 326.233 cm^2^, SE = 28.367 cm^2^; TD = 308.372 cm^2^, SE = 23.521 cm^2^) indicating that between-group differences in VTC (*ω*)_Spatial_ and VTC (*τ*)_Temporal_ minima were not due to differences in the extent to which children with ASD and TDs could lean in any direction.

**Fig. 5:**
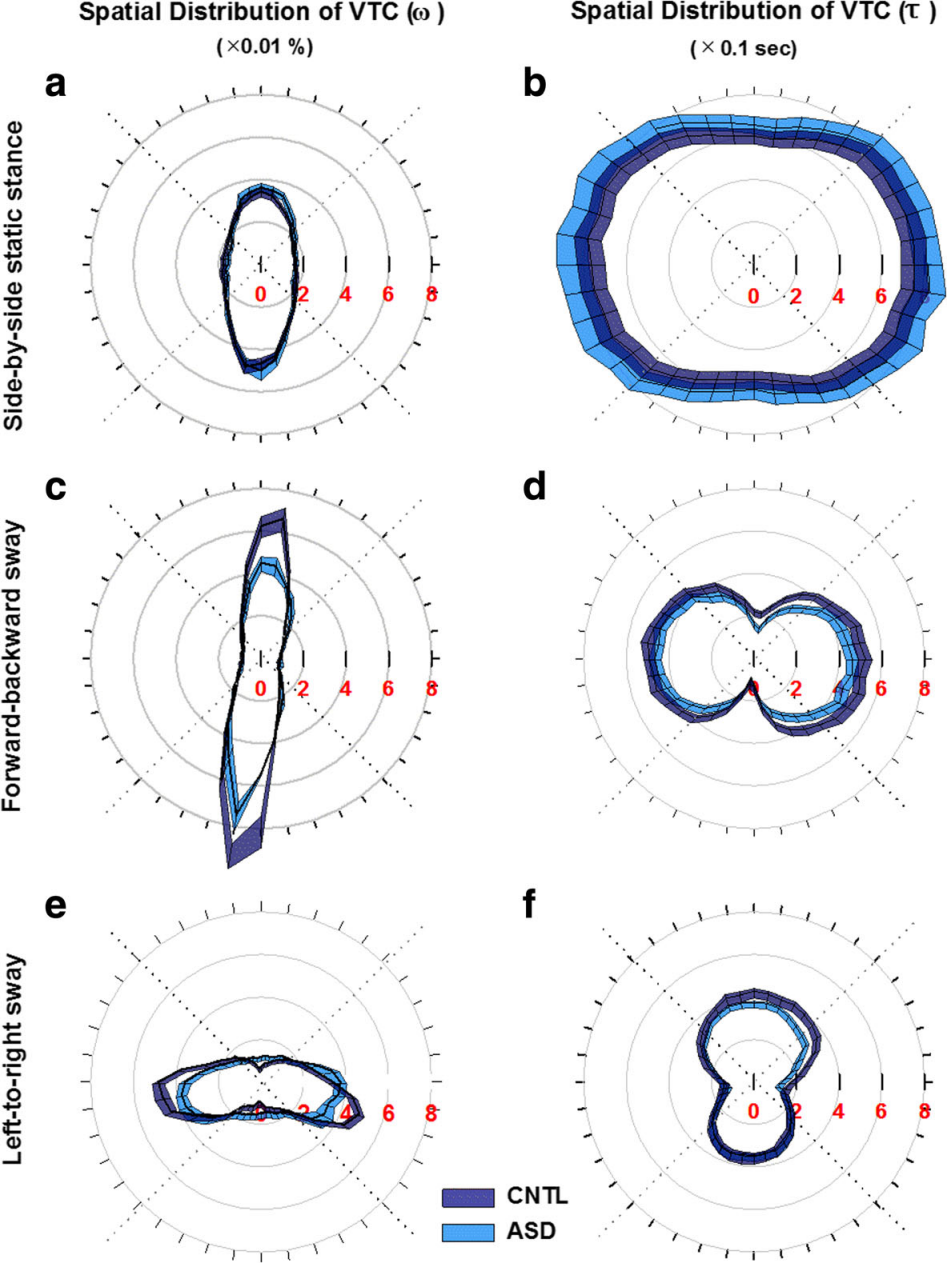
Spatial distribution of VTC (*ω*)_Spatial_ (panels **a**, **c**, **e**) and VTC (*τ*)_Temporal_ minima (panels **b**, **d**, **f**) (mean ± SE) as a function of task condition (side-by-side static stance: panels **a** and **b**; forward-backward sway: panels **c** and **d**; left-to-right sway: panels **e** and **f**). *Red* indices on the *left column* represent the percentage distribution (×0.01%) of VTC (*ω*)_Spatial_ at each postural limitation boundary segment. *Red* indices on the *right column* represent the temporal distribution (×0.1 s) of VTC (*τ*)_Temporal_ minima at each postural limitation boundary segment. *Gray dotted lines* represent quadrant boundaries we defined for statistical analyses. The *black lines* drawn through the middle of the shaded areas of each group represent group means. *Shaded areas* represent the standard error

During static stance, both groups showed increased VTC (*ω*)_Spatial_ distribution in the forward and backward quadrants compared to the leftward and rightward quadrants (quadrant main effect: *F*_3, 164_ = 186.107, *p* = 0.000; forward = 29%, SE = 0.6%; backward = 35%, SE = 0.6%; leftward = 18%, SE = 0.6%; and rightward = 18%, SE = 0.6%) (Fig. 5a). There was no difference in VTC (*ω*)_Spatial_ for children with ASD and TD controls during static stance (group main effect: *F*_1, 164_ = 0.016, *p* = 0.899) at any quadrants (quadrant × group interaction effect: *F*_3, 164_ = 1.142, *p* = 0.334). VTC (*τ*)_Temporal_ minima were greater laterally compared with the backward quadrant for both groups (quadrant main effect: *F*_3, 164_ = 4.101, *p* = 0.008; leftward-backward = 0.155 s, SE = 0.051 s; rightward-backward = 0.147 s, SE = 0.051 s). Children with ASD showed increased VTC (*τ*)_Temporal_ minima during static stance relative to TD controls (group main effect: *F*_1, 164_ = 5.936, *p* = 0.016; ASD-TD = 0.088 s, SE = 0.036 s) (Fig. 5b).

During dynamic AP sway, VTC (*ω*)_Spatial_ showed greater distribution in forward and backward quadrants compared to leftward and rightward quadrants (quadrant main effect: *F*_3, 160_ = 637.329, *p* = 0.000; forward = 32%, SE = 0.6%; backward = 38%, SE = 0.6%; leftward = 10%, SE = 0.6%; rightward = 10%, SE = 0.6%). Children with ASD showed a significant reduction of VTC (*ω*)_Spatial_distribution compared with TD controls (group main effect: *F*_1, 160_ = 19.354, *p* = 0.000; ASD-TD = −2.5%, SE = 0.6%) with the effect more pronounced in forward and backward relative to leftward and rightward directions (quadrant × group interaction effect: *F*_3, 160_ = 6.238, *p* = 0.000; forward, ASD-TD = −5.2%, SE = 1%; backward, ASD-TD = −4.7%, SE = 1.0%; leftward, ASD-TD = 0.8%, SE = 1.0%; and rightward, ASD-TD = 0.6%, SE = 1.0%) (Fig. 5c). For VTC (*τ*)_Temporal_ minima, both groups showed reductions along the target directions relative to the orthogonal directions (quadrant main effect: *F*_3, 160_ = 45.482, *p* = 0.000; forward = 0.290 s, SE = 0.016 s; backward = 0.258 s, SE = 0.016 s; leftward = 0.458 s, SE = 0.016 s; and rightward = 0.461 s, SE = 0.016 s). In contrast to findings from the static stance condition, children with ASD showed a VTC (*τ*)_Temporal_ minima reduction compared with TD controls (group main effect: *F*_1, 160_ = 13.268, *p* = 0.000; ASD-TD = −0.058 s, SE = 0.016 s) suggesting that affected children showed a reduced amount of time to correct their postural sway before it reached their postural limitation boundary (Fig. 5d).

During dynamic ML sway, VTC (*ω*)_Spatial_ showed greater distribution along the leftward and rightward relative to forward and backward quadrants (quadrant main effect: *F*_3, 164_ = 174.356, *p* = 0.000; forward = 11.8%, SE = 0.7%; backward = 16.6%, SE = 0.7%; leftward = 30.1%, SE = 0.7%; and rightward = 30.1%, SE = 0.7%). VTC (*ω*)_Spatial_ distribution was significantly reduced in the leftward quadrant for children with ASD compared to TD children (quadrant × group interaction effect: *F*_3, 164_ = 5.304, *p* = 0.002; leftward, ASD-TD = −5.1%, SE = 1.4%) (Fig. 5e). Both groups showed decreased VTC (*τ*)_Temporal_ minima along the target directions relative to orthogonal directions (quadrant main effect: *F*_3, 164_ = 48.825, *p* = 0.000; forward = 0.380 s, SE = 0.012 s; backward = 0.332 s, SE = 0.012 s; leftward = 0.211 s, SE = 0.012 s; and rightward = 0.212 s, SE = 0.012 s). Children with ASD showed a significant VTC (*τ*)_Temporal_ minima reduction compared with TD controls (group main effect: *F*_1, 164_ = 5.488, *p* = 0.020; ASD-TD = −0.029 s, SE = 0.012 s) (Fig. 5f).

### Mutual information

Mutual information was greater for ML sway compared to other conditions (stance condition main effect: *F*_2, 80_ = 8.544, *p* = 0.000). Children with ASD showed increased levels of mutual information compared to TD children (group main effect: *F*_1, 40_ = 24.253, *p* = 0.000), especially during dynamic stances (stance condition × group interaction effect: *F*_2, 80_ = 10.755, *p* = 0.000; AP sway: ASD-TD = 0.212 bit, SE = 0.048 bit, *p* = 0.000; ML sway: ASD-TD = 0.335 bit, SE = 0.069 bit, *p* = 0.000). Children with ASD showed increased mutual information during ML sway compared to both AP sway and static stance (ML sway–AP sway = 0.140 bit, SE = 0.044 bit, *p* = 0.009; ML sway–static stance = 0.290 bit, SE = 0.053 bit, *p* = 0.000; AP sway–static stance = 0.150 bit, SE = 0.039 bit, *p* = 0.001) whereas TD children showed similar levels of mutual information across all task conditions (Fig. 4c).

### Demographic and clinical correlations

**Fig. 6:**
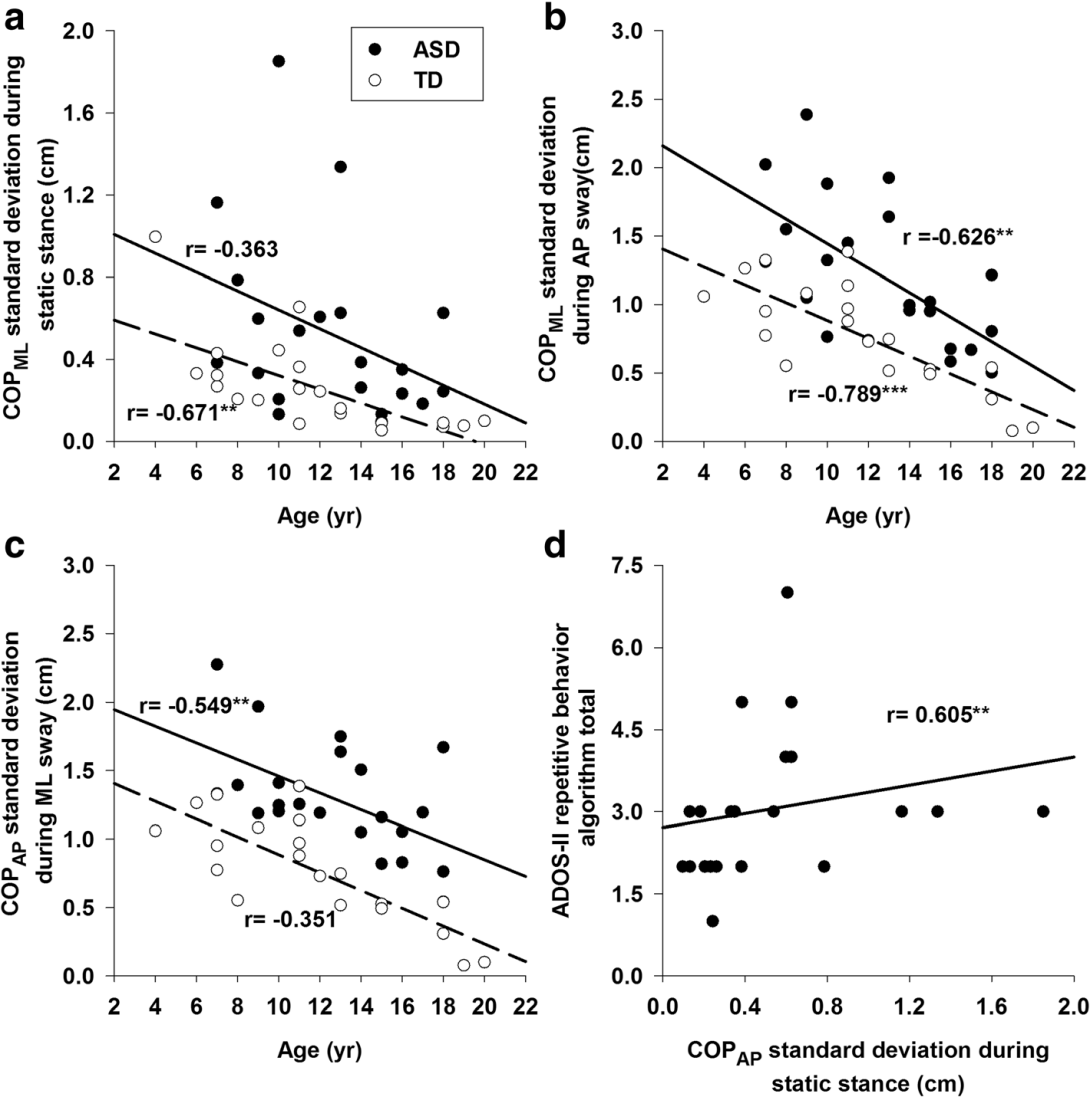
Relationship between postural performance and key demographic and clinical characteristics of participants. **a** Increased age was associated with a COP_ML_ standard deviation reduction in TD children during static stance. **b** Increased age was associated with COP_ML_ standard deviation reductions of both groups during dynamic AP sway. **c** Increased age was associated with a COP_AP_ standard deviation reduction in children with ASD during dynamic ML sway. **d** Increased ADOS-II ratings of restricted, repetitive behavior algorithm total was associated with increased COP_AP_ standard deviation during static stance. Correlation coefficients are marked as **0.01 level and ***0.001 level

None of the postural measurements were associated with IQ scores (i.e., verbal, performance or full-scale IQ) for either group (Table 2). For TD children, increased age was associated with lower COP_ML_ standard deviation and COP trajectory length during static stance (Fig. 6a). Increased age of TD children was also associated with reductions of COP_ML_ standard deviation and mutual information during dynamic AP sway (Fig. 6b). For children with ASD, increased age was associated with reduced COP standard deviation in directions orthogonal to the target during dynamic stances (Fig. 6b, c). The strength of age and postural control associations did not differ between groups (*p* > .05). Increased COP_AP_ standard deviation during static stance was associated with higher clinical ratings of restricted-repetitive behaviors on the ADOS-II for children with ASD (Fig. 6d).

**Table 2:** Correlation coefficients between postural measurements and demographic, cognitive and ASD clinical ratings

TD (*n* = 21)	Age	FSIQ	PIQ	VIQ				
SS_COP_ML_	−0.671**	−0.118	−0.245	−0.108				
SS_COP_AP_	−0.480	−0.123	−0.167	−0.137				
AP_COP_ML_	−0.789***	−0.069	−0.247	0.063				
AP_COP_AP_	−0.057	−0.080	−0.068	−0.050				
ML-COP_ML_	0.395	0.140	0.257	0.104				
ML_COP_AP_	−0.351	0.176	0.054	0.279				
AP_MI	−0.666**	−0.309	−0.324	−0.290				
ML_MI	−0.211	−0.134	−0.116	−0.114				
SS_Length	−0.704***	−0.135	−0.155	−0.171				
AP_Length	0.008	0.216	−0.045	0.299				
ML_Length	0.203	0.286	0.171	0.394				
ASD (*n* = 22)	Age	FSIQ	PIQ	VIQ	ADI-R Social	ADI-R Comm	ADI-R RRB	ADOS RRB
SS_COP_ML_	−0.363	−0.063	−0.089	−0.044	0.066	−0.070	−0.093	0.211
SS_COP_AP_	−0.286	−0.250	−0.360	−0.125	0.085	0.114	−0.288	0.605**
AP_COP_ML_	−0.626**	0.141	0.110	0.149	0.092	−0.038	−0.094	0.138
AP_COP_AP_	0.504	0.085	0.066	0.072	0.183	−0.019	0.211	−0.133
ML-COP_ML_	0.298	−0.184	−0.250	−0.111	0.090	−0.050	0.070	0.347
ML_COP_AP_	−0.549**	0.164	0.055	0.237	−0.060	−0.088	−0.063	0.266
AP_MI	0.212	−0.026	−0.154	0.088	0.457	0.299	0.305	−0.172
ML_MI	−0.328	−0.026	−0.183	0.122	0.130	−0.083	−0.049	0.241
SS_Length	−0.265	−0.125	−0.387	−0.102	0.218	−0.024	−0.151	0.206
AP_Length	−0.297	0.196	0.235	0.126	−0.087	−0.528	−0.057	0.170
ML_Length	−0.498	−0.117	−0.158	−0.050	−0.034	−0.519	−0.310	0.311

*SS_ COP*_*ML*_ COP_ML_ standard deviation of static stance, *SS_ COP*_*AP*_ COP_AP_ standard deviation of static stance, *AP_ COP*_*ML*_ COP_ML_ standard deviation of dynamic AP sway, *AP_ COP*_*AP*_ COP_AP_ standard deviation of dynamic AP sway, *ML_ COP*_*ML*_ COP_ML_ standard deviation of dynamic ML sway, *ML_ COP*_*AP*_ COP_AP_ standard deviation of dynamic ML sway, *AP_MI* mutual information of dynamic AP sway, *ML_MI* mutual information of dynamic ML sway, *SS_Length* COP trajectory length of static stance, *AP_Length* COP trajectory length of dynamic AP sway, *ML_Length* COP trajectory length of dynamic ML sway, *FSIQ* full scale IQ, *PIQ* performance IQ, *VIQ* verbal IQ, *ADI-R Social* ADI-R social algorithm total, *ADI-R Comm* ADI-R verbal communication algorithm total, *ADI-R RRB* ADI-R restricted and repetitive behavior algorithm total, *ADOS-RRB* ADOS-II restricted and repetitive behavior algorithm total

Statistical significance at **α = 0.01 and ***α = 0.001

## Discussion

To our knowledge, this is the first study to examine postural orientation and equilibrium deficits underlying increased postural sway in children with ASD. Unlike the majority of prior studies, we also assessed children’s postural stability during static stance and dynamic stances that more closely resemble the dynamic aspects of everyday activities, including walking and reaching for objects while standing. We utilized both traditional measures of postural control, including COP standard deviation and trajectory length, and novel measures of postural orientation (VTC spatial and VTC temporal) and equilibrium control processes (mutual information) that have been well validated in motor control and biomechanical fields, but never applied to studies of ASD. There are three key findings from this study. First, children with ASD showed increased COP trajectory length and standard deviation during all standing conditions relative to TD controls, but elevations in COP standard deviation shown by children with ASD were more severe during dynamic stances. Second, children with ASD displayed greater VTC (*τ*)_Temporal_ minima during static stance. In the context of their increased COP variability, this suggests that children with ASD compensate for their postural instability by allowing themselves more time to correct their postural sway before it reaches the postural limitation boundary. In contrast, children with ASD were not able to show this same compensatory process during dynamic stances. They also showed significant VTC (*ω*)_Spatial_ reductions indicating that their ability to acquire spatial information regarding their sway is impaired during dynamic but not static stances. Third, increased mutual information of COP_AP_ and COP_ML_time series was observed during dynamic stances in children with ASD suggesting a compromised ability to decouple distinct joint movements when attempting to sway in one direction. Taken together, these results suggest that both postural orientation and equilibrium control processes are disrupted in ASD, but the manifestations of these disruptions vary across different standing conditions.

### Increased postural sway in ASD during static and dynamic stances

Our findings are consistent with prior studies showing increased COP standard deviation and trajectory length in children with ASD during static stance, but extend these results to indicate that increases in COP variability become more severe when children with ASD attempt to sway along specified directions. During dynamic stances, gravitational torque increases relative to static stance due to increases in postural sway magnitude, velocity, and acceleration [24, 25, 40]. The goal of dynamic stances is distinct from static stance in that participants introduce internal perturbations to their postural control system by moving themselves along a target direction and then reactively refine their postural sway velocity and acceleration to avoid contact with their postural limitation boundary. The task becomes more difficult as children approach their postural limitation boundary because the direction of sway acceleration must be reversed and velocity must subsequently return to zero prior to the individual’s COP colliding with the boundary [25, 40]. The increased demands on control processes used to maintain stability despite constant and intentional movement appear to contribute to postural deficits in children with ASD that are more severe than the static stance conditions that have been studied previously.

### Altered postural orientation in ASD during static and dynamic stances

To assess the control processes contributing to increased postural sway variability in ASD, we examined participants’ COP time series relative to their postural limitation boundary. During standing, the postural limitation boundary serves as an internal representation of the maximum extent to which an individual may sway without losing balance [12–14]. In our study of static stance, we did not see any difference in VTC (*ω*)_Spatial_ between groups suggesting that children with ASD have a relatively spared ability to acquire postural sway spatial information relative to their limitation boundary (Fig. 5a). In order to ensure that one’s postural sway does not collide with or move beyond the postural limitation boundary, individuals maintain a “safety margin” from the postural limitation boundary [12, 20]. The safety margin can be preserved by consistently perceiving spatial information of the postural limitation boundary regarding directions to which postural stability may be reduced [23]. Our finding that children with ASD utilize spatial information similar to controls indicates that their ability to preserve a safety margin by dynamically processing spatial information during static stance is intact. The safety margin also can be maintained by reducing the magnitude of postural sway [12–14] and/or increasing the time it will take for the COP to reach the postural limitation boundary (i.e., VTC (*τ*)_Temporal_ minima) [21–23]. VTC (*τ*)_Temporal_ minima was increased in children with ASD compared to TD controls during static stance suggesting that, in the context of increased postural instability, they may utilize a compensatory process that affords them more time to direct their body movement away from their postural limitation boundary despite increased sway and sway variability (Fig. 5b).

During dynamic stances, children with ASD showed reduced VTC (*ω*)_Spatial_ along the target directions compared to TD children indicating that they acquired less spatial information along directions that would potentially induce postural instability (Fig. 5c, e). Children with ASD also showed a decreased VTC (*τ*)_Temporal_ minima suggesting that they have a reduced amount of time to adjust their postural sway away from their postural limitation boundary (Fig. 5d, f). These findings contrast with our results from the static stance condition and suggest that when children with ASD are required to dynamically adjust the velocity and acceleration of their postural sway, they are not able to invoke the same compensatory mechanisms as they do when attempting to stand still. Therefore, our results identify an atypical pattern of postural orientation in ASD that is exacerbated by increased task demands, and which may be more evident during everyday activities involving dynamic shifting of children’s COP (e.g., mediolateral trunk sway during walking, rhythmical arm reaching from one location to the other, rocking in a chair). These findings suggest that studies examining sensorimotor behaviors in ASD may be more informative for treatment development efforts if they focus on dynamic activities similar to those postural activities executed in the context of daily living rather than static postural control tasks that emphasize reducing movement while standing still.

Children with ASD and controls showed postural limitation boundaries that were similar in size suggesting that all confounding variables that could possibly affect participants’ boundary are well controlled (Table 1). It remains possible that postural limitation boundaries may vary in size in ASD when age and key physical characteristics of height and weight are not controlled. By matching groups on these variables in our study, differences in VTC measurements reported among children with ASD can be seen as being more directly reflective of postural orientation deficits rather than differences in physical characteristics.

### Altered postural equilibrium in ASD across dynamic stances

We also found that children with ASD show significant increases in mutual information during dynamic stances implicating deficits in postural equilibrium process (Fig. 4c). Mutual information typically is reduced during dynamic stances compared to static stance as postural sway is constrained within one dimension and one type of joint movement is emphasized over another [41]. In contrast, we found that children with ASD showed increased levels of shared information between ankle and hip movement during dynamic compared to static stances. These findings suggest that failures to decouple ankle dorsi-/plantar-flexion and hip abduction/adduction may contribute to postural disruptions during dynamic stances in ASD. Increased dependency of COP_AP_ and COP_ML_ in ASD may reflect a reduced ability to evoke distinct motor control processes and move towards the target without generating unwanted body movements in orthogonal directions [26, 27].

Alternatively, increased mutual information may suggest a compensatory process that allows children with ASD to increase their body sway generally and actively engage multiple control mechanisms in order to decrease the likelihood that they will lose balance if depending on only one joint action. While this strategy may assist children in realizing the task goal, it also inevitably disrupts their balance as increased variability along directions orthogonal to the target has been shown to compromise an individual’s abilities to maintain stability [42] and realize task goals that involve intentional sway, such as reaching for an object [43].

### Postural stability, developmental and clinical features in ASD

Our findings that COP trajectory length and variability decrease with age in TD children but not children with ASD are consistent with those reported by Minshew et al. [4] who showed that postural stability in children with ASD failed to reach adult levels during development. Children with ASD showed reductions in orthogonal COP variability with age indicating that the ability to independently activate ankle or hip mechanisms during dynamic stances may develop along a delayed timeline, but that equilibrium control processes may continue to mature throughout childhood (Fig. 6b, c). Longitudinal studies assessing postural orientation and equilibrium during dynamic standing activities are needed to characterize the timing and nature of these deficits across the lifespan in ASD.

We also found that increases in COP standard deviation in the AP direction during static stance were associated with increased severity of repetitive behaviors in ASD (Fig. 6d). A similar relationship between reduced postural control and increased repetitive behaviors previously was documented in children with ASD [29, 30] and children with intellectual disability [44] suggesting that common motor control and possibly neurodevelopmental mechanisms may contribute to a broad range of motor abnormalities in these populations. While evidence also exists that confounding factors including age and IQ scores affect the relationship between balance control and repetitive behaviors in ASD as they both commonly present negative correlations with children’s development and cognitive abilities [45], further studies are needed to systematically examine mechanisms underlying this interesting relationship.

### Study limitations and future directions

While the present study documents multiple novel findings useful for developing more mechanistic models of postural control deficits in ASD, our results must be considered in the context of multiple limitations. First, we included children across a broad age range (4–18 years) in order to better characterize postural control processes across childhood in ASD. Still, denser sampling of individual periods of development and longitudinal follow-up studies are needed to map trajectories of postural control development and their relation to clinical symptoms in ASD. Second, we also chose to match our groups on performance as opposed to verbal IQ as we and others have done previously [46–49]. This allowed us to maximize the generalizability of our findings by including children whose verbal ability may be below average despite average nonverbal abilities as is common in ASD [50]. Third, some of the participants with ASD in our sample may have comorbid conditions that are common in this disorder (e.g., ADHD, depression). Systematic study of the relationships between these comorbid conditions and postural control in ASD is needed. Last, while the VTC measurement approaches used here have been well validated in prior studies of aging [23], mild traumatic brain injury [22], ankle instability [19], and Parkinson disease [20], these approaches may have limitations when studied with children with ASD. We controlled for multiple variables that may confound these measurements (e.g., height, weight, age, verbal ability), but it remains possible that performance for some children with ASD or TD children may have been disrupted by factors such as reduced attention, motivation, or understanding of the task. Larger studies assessing the extent to which attention, verbal abilities, and age are related to VTC measurements are warranted.

## Conclusions

Our results demonstrate that postural orientation and equilibrium deficits contribute to reduced postural stability in children with ASD during both static and dynamic stances. Studies of more naturalistic dynamic standing activities in ASD are needed to better define how deficits in orientation and equilibrium control processes affect sensorimotor behaviors performed during daily living. An emerging literature has indicated that early motor developmental abnormalities are among the earliest signs of ASD [2, 11] and, combined with our findings that postural control impairments are associated with age and clinically rated restricted and repetitive behaviors in ASD, these results suggest that studies of the development of postural control in ASD may provide important insights into neurodevelopmental mechanisms that cause ASD and the emergence of sensorimotor and other core symptoms during childhood.

## Additional files

Additional file 1: Supplemental information on virtual time-to-contact (VTC) calculations. (DOCX 112 kb)
Additional file 2: Supplemental information on COP based measurements. (DOCX 124 kb)

## Abbreviation

ADI-R: Autism Diagnostic Inventory-Revised
ADOS-II: Autism Diagnostic Observation Schedule-II
AP: anterior-posterior direction
AP sway: anterior-posterior postural sway (dynamic condition)
ASD: autism spectrum disorder
ML: mediolateral direction
ML sway: mediolateral postural sway (dynamic condition)
TD children: typically developing children

## Glossary

Force: Force is a push or pull on an object. If the net force on an object is not zero, then the object accelerates (or changes its velocity). Force is a vector and has both magnitude and direction. Force recorded from a force platform includes measurements in three dimensions, including anterior-posterior, mediolateral, and vertical directions. Force along the vertical direction is typically referred to the ground reaction force.
Moment: The moment is the turning effect produced by a net force perpendicular to the point of rotation.
COP: The point location of the vertical ground reaction force vector. The COP represents a weighted average of pressures over the surface area (i.e., feet) in contact with the ground. It has been used as an indirect measure of individuals’ postural sway. The COP can be derived from the force and moment data collected from a force platform (Formular1).
COP_AP_: Center of pressure time series in the anterior-posterior direction
COP_ML_: Center of pressure time series in the mediolateral direction
VTC: Virtual time-to-contact, a measurement quantifies the spatiotemporal relation of an individual’s postural sway to his/her own postural limitation boundary
VTC (*ω*)_Spatial_: Spatial virtual time-to-contact, a resultant variable from the VTC measurement characterizing the spatial relation of an individual’s postural sway to his/her own postural sway boundary
VTC (*τ*)_Temporal_: Temporal virtual time-to-contact, a resultant variable from the VTC measurement characterizing the temporal relation of an individual’s postural sway to his/her own postural sway boundary
Mutual information: In probability and information theory, the mutual information of two random variables measures their mutual dependency quantifiing the amount of information obtained about one random variable through the other
