# Supplementary material for: Postural orientation and equilibrium processes associated with increased postural sway in autism spectrum disorder (ASD)
Source: J Neurodev Disord. 2016 Nov 25;8:43. doi: 10.1186/s11689-016-9178-1 (PMC5124312; doi:10.1186/s11689-016-9178-1)
Supplement: Additional file 1: — Supplemental information on virtual time-to-contact (VTC) calculations. (DOCX 112 kb) [file 11689_2016_9178_MOESM1_ESM.docx]

**Supplemental information on virtual time-to-contact (VTC) calculations**

VTC was quantified by examining the spatiotemporal relationship between the COP time series derived from each trial and each child’s own postural limitation boundary. The VTC (ω)_Spatial_ represents the location that a specific COP data point would contact with the postural limitation boundary if the data point were to move from its current location on its real trajectory with its instantaneous initial velocity and constant acceleration. The VTC (τ)_Temporal_ was defined as the time it would take for the same COP data point to reach the specific stability boundary location [21-23].

To calculate VTC (τ)_Temporal_ for each instantaneously measured COP data point, the real time was stopped at a current moment ($t_{i}$) and the virtual motion of the data point with its constant acceleration was simulated. According to Newton’s second law, the resultant force as well as the acceleration $\vec{a}(t_{i})$ were considered to be constant while the COP data point was moving along its virtual trajectory from its initial position $\vec{r}(t_{i})$with its instantaneous initial velocity $\vec{v}(t_{i})$ until it would collide with one of the postural limitation boundary segments (Figure 2A; 40 counter-clockwise labeled segments defined in the current study).

The position vector of the COP data point on the virtual trajectory $\vec{\rho_{i}}(\tau)$ started at the moment $t_{i}$, and the time function was obtained by double integration of the constant acceleration $\vec{a}\left( \tau\right)=\vec{a}\left( t_{i} \right)$ with respect to the virtual time parameter τ:

$\vec{\rho_{i}}\left( \tau\right)=\vec{r_{i}}\left( t_{i} \right)+\vec{v}\left( t_{i} \right)\cdot\tau+\vec{a}(t_{i})\cdot\frac{\tau^{2}}{2}$ (1)

The same equation can be written in terms of *x*(COP_AP_) and *y*(COP_ML_) with respect to the reference frame attached to the force platform as:

$x_{i}\left( \tau\right)=\vec{r_{x}}\left( t_{i} \right)+\vec{v_{x}}\left( t_{i} \right)\cdot\tau+\vec{a_{x}}(t_{i})\cdot\frac{\tau^{2}}{2}$ (2)

and

$y_{i}\left( \tau\right)=\vec{r_{y}}\left( t_{i} \right)+\vec{v_{y}}\left( t_{i} \right)\cdot\tau+\vec{a_{y}}(t_{i})\cdot\frac{\tau^{2}}{2}$ (3)

where $\vec{r_{x}}\left( t_{i} \right)$and $\vec{r_{y}}\left( t_{i} \right)$ are components of instantaneous initial position vector; $\vec{v_{x}}\left( t_{i} \right)$and $\vec{v_{y}}\left( t_{i} \right)$ are components of instantaneous initial velocity vector; $\vec{a_{x}}\left( t_{i} \right)$ and $\vec{a_{y}}\left( t_{i} \right)$ are components of instantaneous initial acceleration vector in the AP and ML directions, respectively.

The VTC (ω)_Spatial_ is labeled by examining the spatial coordinate of the virtual time-to-contact location between the virtual trajectory of each COP data point and the postural limitation boundary. Let ($x_{c}, y_{c}$) denote the coordinate on the boundary where the virtual trajectory contacts with for the first time. Given the fact that we dissected the postural limitation boundary to 40 evenly expanded segments, coordinates of each segment’s end points can be derived. If the end points of the corresponding boundary segment are $(x_{1}, y_{1})$ and $(x_{2}, y_{2})$, the slope of the boundary segment is:

$s=(y_{2}-y_{1})/(x_{2}-x_{1})$ (4)

Assuming a constant slope in the differential segment between $(x_{1},y_{1})$and $(x_{2}, y_{2})$, the slope can also be computed as:

$s=(y_{c}-y_{1})/(x_{c}-x_{1})$ (5)

The components of the position vector for $(x_{c}, y_{c})$ were determined by:

$x_{c}\left( \tau\right)=\vec{r_{x}}\left( t_{i} \right)+\vec{v_{x}}\left( t_{i} \right)\cdot\tau+\vec{a_{x}}(t_{i})\cdot\frac{\tau^{2}}{2}$ (6)

and

$y_{x}\left( \tau\right)=\vec{r_{y}}\left( t_{i} \right)+\vec{v_{y}}\left( t_{i} \right)\cdot\tau+\vec{a_{y}}(t_{i})\cdot\frac{\tau^{2}}{2}$ (7)

Substituting $x_{c}$ and $y_{c}$ from Eqs. (6) and (7) in (5), and equating it to Eq. (1), gives a quadratic equation in τ. For each measured COP data point, this procedure was repeated until time parameters for all possible crossing points of the simulated virtual trajectory with all boundary segments were computed. Then, the minimum positive time parameter τ associated with the first crossing point (i.e., VTC (ω)_Spatial_) was assigned to VTC (τ)_Temporal_. Please note that infinity is a legitimate value for VTC(τ)_Temporal_, which means that contact will never occur. A VTC(τ)_Temporal_ equals to 0 means that the COP data point is in contact with the boundary.
