# Supplementary material for: Postural orientation and equilibrium processes associated with increased postural sway in autism spectrum disorder (ASD)
Source: J Neurodev Disord. 2016 Nov 25;8:43. doi: 10.1186/s11689-016-9178-1 (PMC5124312; doi:10.1186/s11689-016-9178-1)
Supplement: Additional file 2: — Supplemental information on COP based measurements. (DOCX 124 kb) [file 11689_2016_9178_MOESM2_ESM.docx]

**Supplemental information on COP based measurements**

**Demographic and developmental variables potentially affecting children’s postural sway can be considered as covariates in the present study and they include participants’ age, height, weight and IQ scores (i.e., verbal, performance and full scale IQs). We controlled the influences of these variables in our analyses by matching groups as closely as possible on each of these factors (Table 1). Therefore, findings of postural control differences between groups within the manuscript are interpreted as reflective of postural alterations characteristic of ASD, rather than reflecting influences of other possible confounding factors. Still, effects of covariates on key dependent postural satiability measures are reported in this supplemental section. Given the multicollinearity of children’s height and weight, we used body mass index (BMI) as a resultant variable in these statistical models. BMI was calculated using a child’s weight in kilogram divided by the square of height in meter.**

**For COP standard deviation, we conducted a 3 (stance condition: static vs. AP sway vs. ML sway) × 2 (COP direction: AP vs. ML) × 2 (group: ASD vs. TD) fixed effects repeated-measure ANOVA with covariates of age, BMI, verbal, performance and full scale IQs. Consistent with analyses performed without covariates, children with ASD showed greater COP standard deviation as compared to TD controls (F_1,34_=7.892, p=0.008; ASD=2.914 cm, SE=0.133 cm; TD=2.336 cm, SE=0.137cm) and none of the covariates significantly affected the group comparisons. For COP trajectory length and mutual information, two 3 (stance condition) × 2 (group) fixed effects repeated-measure ANOVAs were performed, respectively, with covariates of age, BMI, verbal, performance and full scale IQs. Children with ASD showed greater COP trajectory length (F_1,34_=10.296, p=0.003; ASD=211.274 cm, SE=11.532 cm; TD=151.682 cm, SE=12.579 cm) and mutual information (F_1,35_=20.885, p=0.000; ASD=0.722 bit, SE=0.028 bit; TD=0.522 bit, SE=0.029 bit) compared to controls. The between-group effect of mutual information was affected by children’s age (F_1,35_=4.227, p=0.047) and BMI (F_1,35_=4.879, p=0.034), but remained significant when including these factors in the model.**

**For natural postural sway frequency during AP and ML dynamic sways alone, we compared groups using a 2 (stance condition) × 2 (group) fixed effects repeated-measure ANOVA with the same covariates as described above. Consistent with results reported in the manuscript, there was no difference in sway frequency between individuals with ASD and controls (F_1,35_=0.648, p=0.426; ASD=0.326 Hz, SE=0.019 Hz; TD=0.348 Hz, SE=0.020 Hz).**
